# Thirty-Day Readmission Risk Stratification at Hospital Discharge: Single-Center Temporal Validation and Nursing Implementation Readiness Study

**DOI:** 10.2196/101335

**Published:** 2026-11-09

**Authors:** Hui-Wen Po, Tzu-Yu Huang, Ying-Chien Chu, Hui-Chen Tsai, Chung-Yu Chen, Chien-Hsin Yang

**Affiliations:** 1 Department of Nursing, National Taiwan University Hospital Yunlin Branch, Douliou, Taiwan; 2 Department of Internal Medicine, National Cheng Kung University Hospital, College of Medicine, National Cheng Kung University, Cheng-Hsing Campus, No.1, University Road, Tainan, 701, Taiwan, 886 062353535; 3 Department of Industrial Engineering and Management, Chaoyang University of Technology, Taichung, Taiwan

**Keywords:** nursing informatics, discharge planning, transitional care, clinical decision support, hospital readmission, machine learning, calibration, case management

## Abstract

**Background:**

Discharge-planning nurses and case managers need to prioritize transitional care, but predictive ranking alone does not identify which patients will benefit from a specific intervention.

**Objective:**

This study aimed to develop and temporally validate a local discharge time model for 30-day readmission; evaluate calibration, risk tiers, clinical utility, and subgroup performance; and describe historical nursing action cohorts only as non–model-triggered feasibility evidence for a future pathway.

**Methods:**

This single-center observational study used 41,935 discharge episodes from 2017 to 2018. After excluding 2.3% (964/41,935) in-hospital deaths, 48.5% (19,890/40,971) of episodes from 2017 were used for development, and 51.5% (21,081/40,971) of episodes from 2018 were used for temporal validation. The outcome was the same-institution all-cause readmission within 30 days, defined as the union of readmissions on days 1 to 14 and 15 to 30. Logistic regression, random forest, and Extreme Gradient Boosting were calibrated using 5-fold out-of-fold predictions from 2017. The 95% CIs were obtained with 1000 stratified bootstrap replicates. The separate historical cohorts included shared decision-making (n=72), long-term care linkage (n=364), and remote monitoring (n=43). None was selected or routed by model output.

**Results:**

The validation cohort had 20.7% (4361/21,081) readmissions. Random forest was selected from development cross-validation and achieved an area under the receiver operating characteristic curve (AUROC) of 0.816 (95% CI 0.809-0.823), area under the precision-recall curve (AUPRC) of 0.593 (95% CI 0.579-0.607), and Brier score of 0.119 (95% CI 0.116-0.121); the calibration intercept was –0.117, the slope was 0.974, and the observed-to-expected ratio was 0.951. Readmission occurred in 6.5% (661/10,120) of low-risk episodes, 18.5% (1160/6271) of moderate-risk episodes, 47.3% (1769/3739) of high-risk episodes, and 81.1% (771/951) of very high–risk episodes. The principal diagnosis group accounted for 84.6% of the random forest importance. After excluding index episodes labeled as planned admission or discharge and refitting, the AUROC decreased to 0.726, and the AUPRC decreased to 0.284. In historical cohorts, discharge direction changed in 41.7% (30/72) of cases; 82.7% (301/364) of patients used long-term care; 90.2% (268/297) of patients with valid dates started service within 7 days; and the uncontrolled remote monitoring cohort showed exploratory 3-month reductions in hospital days, emergency visits, and readmissions.

**Conclusions:**

The recalculated local model showed useful temporal discrimination and near-unity calibration after probability calibration, but its performance depended heavily on diagnosis coding and potentially planned readmissions. External generalizability and model actionability remain unestablished. Prospective silent-run evaluation, local threshold review, subgroup monitoring, and controlled testing of the nurse-led response are required before clinical activation.

## Introduction

Thirty-day readmission remains an important indicator of discharge quality and postacute care transitions, and reducing avoidable readmission is a continuing health system priority [1,2]. Discharge-planning nurses and case managers coordinate patient and caregiver education, medication reconciliation, postacute referrals, and postdischarge escalation. Recent international syntheses indicate that transitional care can reduce health care use, but effectiveness varies by population, intervention complexity, follow-up duration, and implementation fidelity [3,4]. Hospitals, therefore, need transparent ways to prioritize limited nursing resources rather than delivering the most intensive intervention to every patient.

Readmission models frequently achieve only modest discrimination and may not translate into useful bedside decisions [5,6]. Earlier transitional care reviews similarly showed heterogeneous intervention effects [7,8]. Recent work suggests that nursing data can contribute predictive information beyond administrative variables [9]. Predictive performance, however, is distinct from clinical actionability: a model may rank patients by risk without identifying who will respond to shared decision-making, long-term care linkage, remote monitoring, or another care component.

Our institutional program previously examined discharge-planning effectiveness, nursing care models, remote health monitoring, and shared decision-making with long-term care linkage [10-13]. These studies describe potentially feasible care components, but they do not constitute a model-triggered intervention pathway. The present study, therefore, treats those cohorts only as historical, illustrative evidence of feasibility and not as proof that the prediction model improves outcomes.

Prediction model guidance emphasizes transparent data sources, predictor timing, outcome definitions, validation, calibration, and applicability [14-16]. For nursing informatics, a further concern is that functional status, caregiver capacity, health literacy, self-management ability, symptom burden, and nursing complexity may be documented in structured assessments, standardized nursing terminologies, or narrative notes rather than administrative fields. Natural language processing and standardized nursing concepts offer possible methods for recovering this information [17-20].

This study aimed to (1) develop and temporally validate a single-center discharge time model for same-institution 30-day readmission; (2) evaluate probability calibration, risk tier separation, decision curve net benefit, subgroup performance, and sensitivity to outcome and cohort definitions; and (3) describe historical nursing action cohorts as illustrative evidence for designing a future, prospectively evaluated workflow. We did not test whether model-triggered transitional care improved patient outcomes.

## Methods

### Study Design and Setting

We conducted a multicomponent observational study at National Taiwan University Hospital Yunlin Branch. The primary component was the development of a prediction model using discharge episodes from 2017, with temporal validation on 2018 episodes. Historical shared decision-making, long-term care linkage, and remote monitoring datasets were analyzed separately to describe the available nursing services. The model was not visible to clinicians, did not alter care, and did not trigger enrollment in any historical cohort. The action cohorts were not used for model training, calibration, model selection, threshold derivation, or treatment effect estimation.

### Prediction Cohort and Outcome

The source extract contained 41,935 discharge episodes. We excluded 964 episodes ending in in-hospital death, leaving 40,971 episodes for analysis: 19,890 in 2017 and 21,081 in 2018. All age groups were retained. The deidentified extract did not contain a stable patient identifier. Therefore, unique patient counts could not be reconstructed, and repeated discharge episodes were treated as independent observations (Table S1 in Multimedia Appendix 1).

The primary outcome was same-institution all-cause readmission within 30 days after discharge. The raw dataset contained separate indicators for readmission on days 1 to 14 and days 15 to 30; the cumulative 30-day outcome was defined as either indicator being positive. The extract did not capture readmissions to other hospitals and did not provide a reliable indicator distinguishing planned from unplanned subsequent admissions. Accordingly, potentially planned readmissions were included in the primary outcome.

During revision, an audit of the raw readmission fields identified that the previously reported 9.7% reflected only days 15 to 30 rather than cumulative 30-day readmission. The outcome was reconstructed as the logical union of the 2 windows, and all downstream analyses were rerun. The revised outcome definition, analysis code, and outputs were independently reviewed and verified by the authors and study statistician (Table S1 in Multimedia Appendix 1).

Postdischarge deaths were not treated as censoring events because the extract did not support time-to-event modeling with complete competing risk data. Among the analytic episodes, 233 deaths occurred within 30 days after discharge; 105 had a recorded readmission before death, and 128 did not. Episodes without a recorded readmission were classified as nonevents. Sensitivity analyses excluded these deaths and used a composite of readmission or death within 30 days.

### Candidate Predictors and Missing Data

Candidate predictors were restricted to information available at discharge: age band, sex, length of stay, broad principal diagnosis group, and discharge destination group. Age was categorized as 0 to 17 years, 18 to 39 years, 40 to 59 years, 60 to 79 years, or 80 years or older. Principal diagnosis labels that changed between calendar years were harmonized into clinically broad groups, including a planned admission or discharge category. Destination indicators and raw disposition codes were combined into the following categories: home, facility, acute transfer, community service, other, or missing. The putative prior readmission field was not modeled because it was blank in 99.9% (40,931/40,971) of the episodes and could not be interpreted reliably.

Age was missing in 0.0% (1/40,971) of episodes, length of stay was missing in 2.2% (887/40,971), and discharge destination was missing in 6.3% (2566/40,971); sex and diagnosis group were complete after harmonization. Categorical missingness was represented explicitly. Length of stay was capped at the 99th percentile in 2017 (54 days), transformed as log(1 + length of stay), and median imputed within the modeling pipeline. Variable definitions are reported in Table S2 in Multimedia Appendix 1, and period-level missingness is reported in Table S1 in Multimedia Appendix 1.

### Model Development, Calibration, and Temporal Validation

We evaluated prespecified logistic regression, random forest, and Extreme Gradient Boosting models. Categorical variables were one-hot encoded. Categories occurring fewer than 5 times were grouped by the encoder. Hyperparameters were fixed before examining the 2018 validation outcomes and are reported in Table S3 in Multimedia Appendix 1. Five-fold stratified cross-validation within 2017 generated out-of-fold predictions. Logistic calibration (Platt scaling) was fitted to these out-of-fold predictions and then applied to predictions from each model refitted on all 2017 episodes.

The candidate model with the highest 2017 out-of-fold area under the precision-recall curve (AUPRC) was selected; Brier score and area under the receiver operating characteristic curve (AUROC) were secondary criteria. The untouched 2018 cohort was used for temporal validation. Performance was summarized using the AUROC, AUPRC, Brier score, calibration intercept, calibration slope, mean predicted risk, observed-to-expected ratio, and a decile calibration plot [14-16] (Table S4 and Figure S1 in Multimedia Appendix 1). The 95% CIs for the AUROC, AUPRC, and Brier score were calculated using 1000 stratified nonparametric percentile bootstrap replicates with seed 20260801. The existing institutional rule-based high-risk flag was retained as a comparator and evaluated in the temporal validation cohort.

Decision curve analysis estimated the net benefit across risk thresholds from 0.05 to 0.50. Because this was a retrospective decision analytic assessment and the score triggered no care, net benefit was interpreted as providing potential decision support rather than as demonstrating clinical effectiveness (Figure S2 in Multimedia Appendix 1).

### Operational Risk Tiers

Four provisional tiers were derived from the calibrated 2017 out-of-fold random forest distribution: below the 50th percentile, from the 50th to below the 80th percentile, from the 80th to below the 95th percentile, and the 95th percentile or higher. The resulting cutoff points (0.1256, 0.3014, and 0.7746) were applied without re-estimation to 2018. Intervals were lower inclusive and upper exclusive except for the low tier, which included probabilities below 0.1256, and the very high tier, which included probabilities of 0.7746 or higher. The tiers were designed for workload planning, not as validated treatment thresholds (Table S5 in Multimedia Appendix 1).

### Subgroup and Sensitivity Analyses

For the selected random forest, we calculated the AUROC, AUPRC, Brier score, observed-to-expected ratio, and calibration slope within age (<65 vs ≥65 years), sex, diagnosis group, and discharge destination subgroups when sample size and event counts permitted. Sensitivity analyses excluded postdischarge deaths within 30 days, used a readmission-or-death composite, restricted the cohort to adults, and excluded index episodes labeled as planned admission or discharge. The fixed-model analysis applied the full cohort model unchanged to the restricted 2018 subset; the refitted analysis re-estimated the random forest and its calibration using the corresponding restricted 2017 subset. This index diagnosis exclusion is an imperfect proxy for potentially planned readmissions, not a direct classification of whether a subsequent admission was scheduled. Random forest feature importance was summarized descriptively, with full subgroup and sensitivity results reported separately (Tables S6-S9 in Multimedia Appendix 1).

### Historical Nursing Action Cohorts and Data Provenance

Three historical datasets were used only to illustrate candidate components for a future nurse-led pathway. No patient was selected or routed to these services by the prediction model. The supplied 72-case shared decision-making dataset and 364-case long-term care dataset match the sample sizes and key outcomes reported by Huang et al [13]. They are therefore treated as overlapping study arms rather than independent evidence. Their incremental contribution here is limited to corrected descriptive denominators and explicit placement within a proposed, not-yet-implemented workflow, consistent with guidance on overlapping publication [21] (Table S10 in Multimedia Appendix 1).

The supplied remote monitoring workbook contained 43 patients enrolled between November 12, 2020, and November 10, 2021, with 3-, 6-, and 12-month use windows. It appears to be an updated or overlapping program dataset compared with the previously published cohort [11], but no patient-level crosswalk was available to quantify the overlap. It is therefore presented as historical program data and not as an independent replication.

Remote monitoring outcomes were paired count measures and were compared using 2-sided Wilcoxon signed-rank tests. No multiplicity adjustment was applied. All *P* values are exploratory. The uncontrolled pretest-posttest design is vulnerable to regression to the mean, secular trends, and confounding by indication and does not support causal claims (Table S11 in Multimedia Appendix 1).

### Proposed Future Nursing Workflow

The proposed pathway separates retrospective model evaluation from future clinical use. Phase 1 is a prospective silent run in which predictions remain hidden and do not change care. The hospital information system would log data availability, missingness, scores, tiers, outcomes, drift, subgroup performance, and projected nursing workload. Only after prespecified technical, calibration, equity, and operational review should activation be considered. In any subsequent activation, the score should prompt structured nursing assessment and shared decision-making rather than automatically determining discharge placement or service enrollment [22] (Table S12 in Multimedia Appendix 1).

### Statistical Analysis of Historical Cohorts

Continuous variables were summarized as means with SDs or medians with IQRs, as appropriate; categorical variables were summarized as counts and percentages. Long-term care initiation times were calculated only when service use and valid nonnegative discharge-to-service dates were available. Remote monitoring means were retained for comparability with the program dataset, while Wilcoxon signed-rank tests were used for paired inference.

### Ethical Considerations

This study was approved by the Research Ethics Committee of National Taiwan University Hospital Yunlin Branch (202403148RINE). Because routinely collected clinical data and retrospectively assembled cohorts were used, the requirement for informed consent was waived. Patients and the public were not involved in the design, conduct, reporting, or dissemination planning of this retrospective analysis. Future prospective deployment should include patient and caregiver feedback on risk communication, shared decision-making, and the acceptability of remote monitoring.

## Results

### Cohort Flow and Characteristics

Of 41,935 source discharge episodes, 964 in-hospital deaths were excluded, leaving 40,971 analytic episodes. Mean age was 62.2 (SD 17.9; range 0-105) years, 57.2% (23,425/40,971) of the episodes involved male patients, and median length of stay was 5 (IQR 3-9) days. The cumulative same-institution 30-day readmission rate was 20.8% (8536/40,971), comprising 4465 events on days 1 to 14 and 4071 events on days 15 to 30. The 2017 development cohort contained 21.0% (4175/19,890) of the events. The 2018 validation cohort contained 20.7% (4361/21,081) of the events (Table S1 in Multimedia Appendix 1).

### Model Performance and Calibration

Random forest achieved the highest development out-of-fold AUPRC and was selected before temporal validation. In 2018, it achieved an AUROC of 0.816 (95% CI 0.809-0.823), an AUPRC of 0.593 (95% CI 0.579-0.607), and a Brier score of 0.119 (95% CI 0.116-0.121; Table 1). Extreme Gradient Boosting had a slightly higher AUROC but lower AUPRC, while logistic regression was less discriminative. The existing rule-based flag had an AUROC of 0.527; readmission occurred in 24.5% (1181/4817) of flagged episodes and 19.6% (3180/16,264) of unflagged episodes (Table 1).

**Table 1.** Temporal validation performance of candidate models^a^.

Models^b^	AUROC^c^ (95% CI)	AUPRC^d^ (95% CI)	Brier score (95% CI)
Random forest	0.816 (0.809-0.823)	0.593 (0.579-0.607)	0.119 (0.116-0.121)
Extreme Gradient Boosting	0.817 (0.810-0.824)	0.589 (0.575-0.604)	0.119 (0.116-0.121)
Logistic regression	0.806 (0.798-0.813)	0.541 (0.528-0.557)	0.121 (0.119-0.124)

a 95% CIs for the 3 statistical models were calculated using 1000 stratified nonparametric percentile bootstrap replicates. The binary rule-based flag was included as an operational comparator; a bootstrap CI was not calculated for that fixed flag.

b Existing rule-based flag: AUROC=0.527, AUPRC=0.217, and Brier score=0.323.

c AUROC: area under the receiver operating characteristic curve.

d AUPRC: area under the precision-recall curve.

The random forest calibration intercept was –0.117, the calibration slope was 0.974, and the observed-to-expected ratio was 0.951; the mean predicted risk was 21.8% (SD 22.2%) compared with an observed rate of 20.7% (4361/21,081). The calibration plot is provided in Figure S1 in Multimedia Appendix 1. Decision curve analysis showed greater net benefit than treat-all and treat-none strategies across thresholds from 0.05 to 0.50, but this retrospective analysis does not establish benefit from actual clinical use (Figure S2 in Multimedia Appendix 1).

### Operational Risk Tiers

Applying development-derived probability cutoff points to the 2018 cohort produced a steep readmission gradient (Table 2). The validation distribution differed modestly from the target 50%, 30%, 15%, and 5% development proportions, as expected in temporal validation. These tiers describe risk separation and projected workload; they were not used to assign care (Table S5 in Multimedia Appendix 1).

**Table 2.** Random forest risk tiers in the 2018 temporal validation cohort^a^.

Tier	Validation^b^, n (%)	Readmission, n/N (%)	Calibrated score interval	Illustrative future nursing action
Low	10,120 (48.0)	661/10,120 (6.5)	<0.1256	Standard education and routine follow-up
Moderate	6271 (29.7)	1160/6271 (18.5)	0.1256 to <0.3014	Nurse follow-up and medication reconciliation
High	3739 (17.7)	1769/3739 (47.3)	0.3014 to <0.7746	Case management review; assess SDM^c^, home care, or long-term care
Very high	951 (4.5)	771/951 (81.1)	≥0.7746	Multidisciplinary review; assess intensive follow-up or facility needs

a Cutoff points were the 50th, 80th, and 95th percentiles of calibrated 2017 out-of-fold random forest predictions. Intervals are lower inclusive and upper exclusive except the low tier and the very high lower bound shown above. Actions are illustrative and were not delivered according to model output.

b Validation-cohort percentages use all 21,081 discharge episodes as the denominator (N=21,081).

c SDM: shared decision-making.

### Feature Importance, Subgroups, and Sensitivity Analyses

The principal diagnosis group accounted for 84.6% of the total random forest impurity importance, followed by length of stay (8.6%), age band (4.5%), sex (1.2%), and discharge destination (1.2%). The planned admission or discharge indicator alone accounted for 62.5% of the total importance. In validation, 74.0% (1783/2411) of the episodes in the planned category had a recorded 30-day return (Table S6 in Multimedia Appendix 1).

Subgroup performance was heterogeneous. Random forest AUROC was 0.861 among patients younger than 65 years and 0.751 among those aged 65 years or older. It was 0.827 in female episodes and 0.807 in male episodes. By discharge destination, AUROC was 0.825 for home discharge but 0.539 for facility discharge. Diagnosis-specific AUROC values were generally modest despite substantial overall discrimination, consistent with strong separation between diagnosis categories rather than uniformly strong within-category ranking. Full subgroup results are reported in Tables S7 and S8 in Multimedia Appendix 1.

Excluding index episodes labeled as planned admission or discharge and refitting the random forest reduced the validation sample to 18,670 episodes with 2578 (13.8%) readmissions. AUROC decreased to 0.726, AUPRC decreased to 0.284, and the calibration slope was 0.936. Results were materially unchanged when postdischarge deaths were excluded, when readmission or death was used as a composite, or when analysis was restricted to adults (Table S9 in Multimedia Appendix 1).

### Historical Nursing Action Cohort Outcomes

Selected historical nursing cohort outcomes not triggered by the model are listed in Table 3. In the overlapping shared decision-making dataset, discharge direction changed in 41.7% (30/72) of cases, the recommended and actual directions were concordant in 59.7% (43/72) of cases, and 15.3% (11/72) of patients had any readmission within 30 days. In the overlapping long-term care dataset, 82.7% (301/364) of patients used long-term care services. Among 297 users with valid nonnegative service dates, the median time to service initiation was 1 (IQR 0-5) day, 66.7% (198/297) initiated within 3 days, and 90.2% (268/297) initiated within 7 days (Table S13 in Multimedia Appendix 1).

**Table 3.** Selected historical nursing cohort outcomes not triggered by the model^a^.

Historical cohort and outcome	Result^b^	Interpretation
Shared decision-making dataset		
Discharge direction changed	30/72 (41.7%)	Previously reported arm; illustrative only
Any 30-d readmission	11/72 (15.3%)	Union of 1-14–d and 15-30–d indicators
Long-term care dataset		
Used long-term care services	301/364 (82.7%)	Previously reported comparison arm
Long-term care users with valid dates		
Service started within 7 d	268/297 (90.2%)	Median 1 (IQR 0-5) d
Remote monitoring		
3-mo hospital days^c^	Mean of 9.49 (SD 14.78) before enrollment to mean of 3.51 (SD 9.63) after enrollment (*P*=.02)	Exploratory Wilcoxon comparison
3-mo emergency visits^c^	Mean of 0.65 (SD 0.69) before enrollment to mean of 0.19 (SD 0.55) after enrollment (*P*=.001)	Exploratory Wilcoxon comparison
3-mo readmissions^c^	Mean of 0.58 (SD 0.59) before enrollment to mean of 0.28 (SD 0.63) after enrollment (*P*=.02)	Exploratory Wilcoxon comparison

a The 72- and 364-case datasets overlap with the study by Huang et al [13]. The 43-patient remote monitoring dataset appears updated or overlapping with the study by Po et al [11], but patient-level overlap could not be quantified. No historical cohort was selected or routed using model scores.

b Results are presented as n/N (%) for the shared decision-making and long-term care datasets and as means, SDs, and *P* values for the remote monitoring dataset.

c Mean changes from before to after enrollment (SD) were −5.98 (17.57) for 3-month hospital days, −0.47 (0.83) for 3-month emergency visits, and −0.30 (0.77) for 3-month readmissions.

In the 43-patient remote monitoring dataset, 3-month hospital days decreased from a mean of 9.49 (SD 14.78) before enrollment to 3.51 (SD 9.63) after enrollment (mean change –5.98; *P*=.02), emergency visits decreased from a mean of 0.65 (SD 0.69) to 0.19 (SD 0.55) (mean change –0.47; *P*=.001), and readmissions decreased from a mean of 0.58 (SD 0.59) to 0.28 (SD 0.63) (mean change –0.30; *P*=.02), while outpatient visits increased from a mean of 0.88 (SD 1.42) to 1.60 (SD 1.56) (mean change +0.72; *P*=.02). At 6 months, emergency visits and readmissions remained lower, whereas hospital days did not differ significantly. At 12 months, the differences in emergency visits and hospital days were not statistically significant. Because no multiplicity correction or concurrent control group was used, these findings represent only exploratory associations (Table 3 and Table S11 in Multimedia Appendix 1).

### Proposed Implementation Pathway

Figure 1 shows the proposed 2-phase pathway. A prospective silent run, technical audit, calibration, subgroup review, and nursing workload assessment precede any activation. If activation is approved later, model output should initiate structured nursing review and shared decision-making rather than automatically determining the discharge destination or service enrollment (Table S12 in Multimedia Appendix 1).

**Figure 1.**
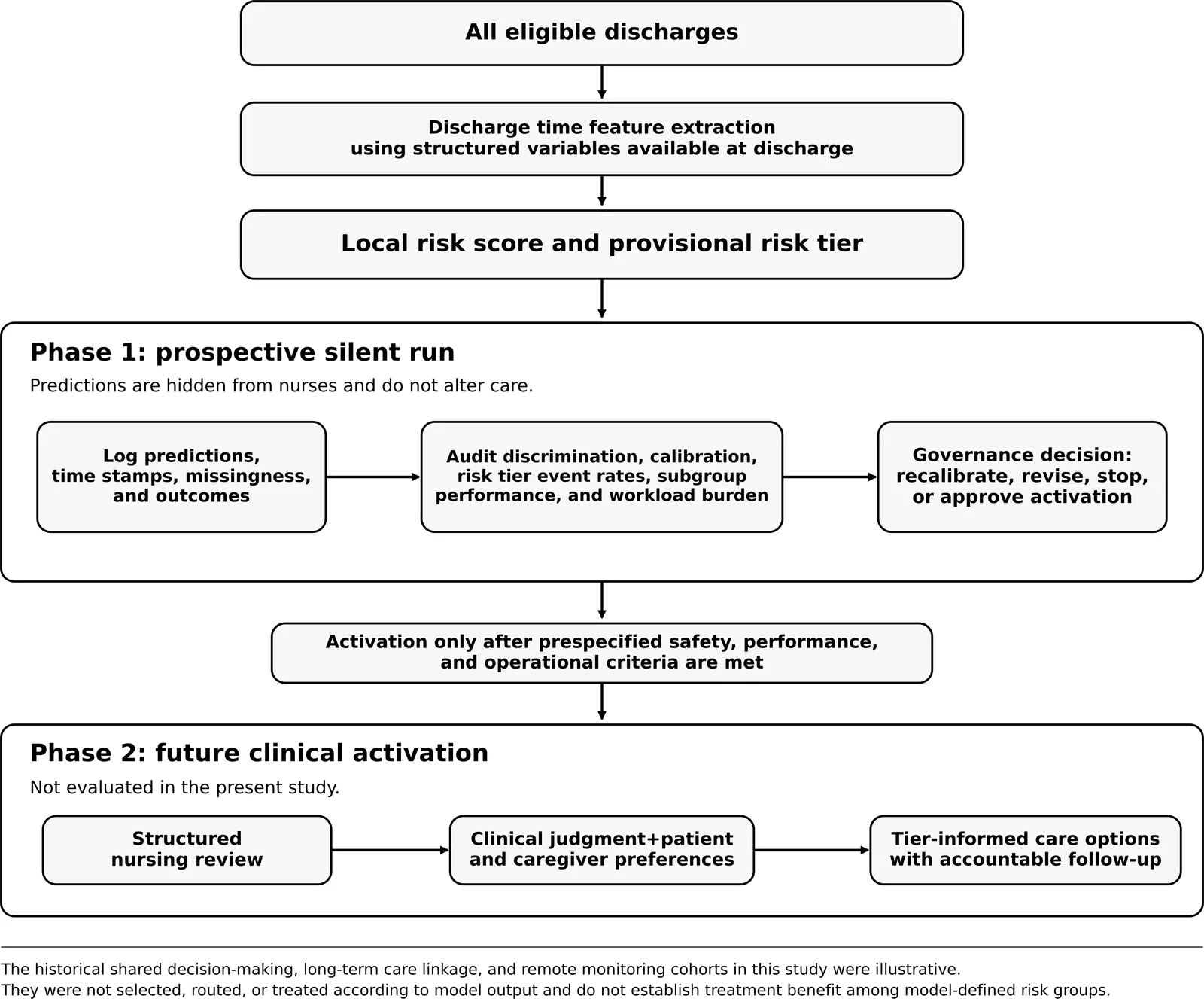
Proposed staged nursing-facing discharge risk workflow. Historical nursing action cohorts are illustrative and are not triggered by the model. Phase 1 is a prospective silent run and phase 2 represents future clinical activation only after prespecified safety, performance, equity, and operational criteria are met.

## Discussion

### Principal Findings

This recalculated single-center study has 4 main findings. First, the corrected cumulative 30-day outcome occurred in approximately one-fifth of discharge episodes rather than the previously reported 9.7%, which represented only the window of days 15 to 30. Second, a calibrated random forest showed useful temporal discrimination, precision-recall performance, and near-unity calibration. Third, the overall performance and tier gradient were driven heavily by principal diagnosis, particularly index episodes labeled as planned admission or discharge. Discrimination decreased substantially when this category was excluded. Fourth, the historical nursing cohorts described available services but did not demonstrate that the model identified patients who benefited from those services.

A model can identify patients with a higher probability of readmission without identifying which intervention, if any, will reduce that probability. Demonstrating actionability requires the prospective linkage of model output to a predefined nursing assessment and care pathway, the measurement of treatment delivery and workload, and the comparison with an appropriate control strategy. The historical cohorts supply only feasibility information for designing that future evaluation.

Probability calibration was acceptable after Platt scaling, and decision curve analysis suggested potential net benefit across a broad range of thresholds. Nevertheless, the high readmission rate in the very high tier should not be interpreted as evidence that intensive transitional care will reduce events. The outcome included potentially planned returns, and the cutoff points were distribution based rather than derived from treatment effects or cost-effectiveness. Before activation, thresholds should be reviewed against contemporary event rates, nurse capacity, intervention eligibility, and the relative harms of missed and unnecessary escalation.

The dominance of the diagnosis group is both a performance signal and a vulnerability. It indicates that differences between broad diagnostic pathways explained much of the observed risk, but it also makes the model sensitive to coding changes, planned care patterns, and case mix shifts. The decrease in performance after excluding this index diagnosis category demonstrates sensitivity to index admission case mix; it does not establish performance for confirmed unplanned subsequent readmissions. Lower performance among older patients and facility discharges also supports prospective subgroup monitoring and caution against a single unexamined threshold for all populations.

The current model omits many determinants of successful care transitions. Recent research on readmissions using nursing data suggests that nursing assessments may provide useful predictive information [9]. Narrative documentation may contain functional limitations, caregiver concerns, self-management barriers, symptom burden, education needs, and home care feasibility that are not visible in administrative coding. Natural language processing and terminology mapping can extract and standardize such activities, although methods and performance reporting remain heterogeneous [17,18].

Health literacy deserves particular attention. Prospective multicenter evidence indicates that inadequate health literacy is associated with readmissions and emergency department visits, especially among patients with high nursing complexity [19]. Standardized nursing diagnoses may also provide clinically interpretable predictors; a recent random forest study ranked individual nursing diagnoses by relevance to intensive care transfer [20]. Future refinement should evaluate structured nursing diagnoses, nursing complexity, health literacy, caregiver burden, functional dependency, discharge readiness, and medication self-efficacy.

No valid external validation was available, and generalizability beyond the development hospital has not been established. We removed the previously proposed University of California, Irvine (UCI) diabetes benchmark because its population, era, feature definitions, and outcome structure were too mismatched to support a clinically meaningful external validation claim. A future external study should use contemporaneous, clinically comparable data with harmonized outcome definitions, handling of planned readmissions, complete calibration assessment, and local recalibration as needed.

A future prospective study should begin with a silent run. Technical reliability, data latency, missingness, calibration, subgroup performance, case manager capacity, review time, alert burden, overrides, and care bundle completion should be measured before scores are shown to clinicians. Early-stage live evaluation should follow relevant clinical AI guidance, including DECIDE-AI (Developmental and Exploratory Clinical Investigations of Decision Support Systems Driven by Artificial Intelligence) [23]. Any subsequent randomized evaluation of an AI-triggered care bundle should follow the CONSORT-AI (Consolidated Standards of Reporting Trials–Artificial Intelligence) [24] guidelines.

### Limitations

This study has substantial limitations. It is a single-center retrospective development and temporal validation study without external validation. The outcome captured only same-institution returns and could not distinguish between planned and unplanned readmissions. Therefore, it is not equivalent to avoidable readmissions. Patient identifiers were unavailable, so repeated episodes were treated as independent, and patient-level clustering could not be modeled. Postdischarge deaths were retained as nonreadmissions when no prior return was recorded, although sensitivity analyses were stable. The feature set was parsimonious; diagnosis dominated; and excluded functional, social, caregiver, medication, physiological, and nursing assessment data. The risk tiers were based on score quantiles and have not been linked to treatment benefit or resource optimization. Subgroup results were retrospective, and some categories were small. The historical nursing datasets overlap with prior publications, are not linked to model scores, and do not constitute independent or causal evidence. The remote monitoring analysis was small, uncontrolled, and susceptible to regression to the mean and confounding by indication and involved multiple exploratory comparisons without multiplicity adjustment.

### Conclusions

A calibrated discharge time random forest stratifies discharge episodes according to their predicted risk of readmission to the same hospital within 30 days after discharge, but the apparent performance depended heavily on diagnosis coding and potentially planned returns. Current evidence supports retrospective model evaluation and implementation planning but not external generalizability, automated care assignment, or benefit from model-triggered nursing interventions. A prospective silent run, explicit ascertainment of planned vs unplanned subsequent readmissions, subgroup and workload monitoring, local threshold review, and a later controlled evaluation of the nurse-led response are prerequisites for clinical activation.

## Supplementary material

10.2196/101335 Multimedia Appendix 1 Supplementary methods, tables, and figures describing 30-day readmission model development and temporal validation, subgroup and sensitivity analyses, historical nursing cohort outcomes, and a proposed implementation monitoring framework.

## Abbreviations

AUPRC: area under the precision-recall curve
AUROC: area under the receiver operating characteristic curve
CONSORT-AI: Consolidated Standards of Reporting Trials–Artificial Intelligence
DECIDE-AI: Developmental and Exploratory Clinical Investigations of Decision Support Systems Driven by Artificial Intelligence
UCI: University of California, Irvine
