# Supplementary material for: Thirty-Day Readmission Risk Stratification at Hospital Discharge: Single-Center Temporal Validation and Nursing Implementation Readiness Study
Source: JMIR Nurs. 2026 Nov 9;9:e101335. doi: 10.2196/101335 (PMC13747315; doi:10.2196/101335)
Supplement: Supplementary file 1 [file nursing-v9-e101335-s001.docx]

Multimedia Appendix 1

# Table S1. Cohort Flow, Outcomes, and Missingness

| **Cohort** | **N** | **Days 1-14 events** | **Days 15-30 events** | **Any 30-day event** | **Role** |
| --- | --- | --- | --- | --- | --- |
| Source extract | 41,935 | 4,468 | 4,071 | 8,539 | Includes 964 in-hospital deaths |
| Excluded in-hospital deaths | 964 | 3 | 0 | 3 | Discharge status coded as death |
| Analytic cohort | 40,971 | 4,465 | 4,071 | 8,536 (20.8%) | All age groups |
| 2017 development | 19,890 | 2,165 | 2,010 | 4,175 (21.0%) | Model development and calibration |
| 2018 temporal validation | 21,081 | 2,300 | 2,061 | 4,361 (20.7%) | Untouched temporal validation |

**Note:** The raw days 1-14 and days 15-30 indicators were combined to form the cumulative 30-day outcome. The prior manuscript incorrectly described the 9.7% days 15-30 rate as cumulative 30-day readmission.

| **Cohort** | **Age missing** | **Sex missing** | **Length of stay missing** | **Diagnosis missing** | **Destination missing** |
| --- | --- | --- | --- | --- | --- |
| Analytic | 1 (0.00%) | 0 (0.00%) | 887 (2.2%) | 0 (0.00%) | 2566 (6.3%) |
| Development | 0 (0.00%) | 0 (0.00%) | 887 (4.5%) | 0 (0.00%) | 1261 (6.3%) |
| Validation | 1 (0.00%) | 0 (0.00%) | 0 (0.0%) | 0 (0.00%) | 1305 (6.2%) |

# Table S2. Variable Definitions and Preprocessing

| **Variable** | **Source definition** | **Analytic handling** |
| --- | --- | --- |
| Outcome | Same-institution readmission within 30 days | Union of raw days 1-14 and days 15-30 indicators; planned and unplanned returns not distinguishable |
| Age band | Age at discharge | 0-17, 18-39, 40-59, 60-79, and >=80 years; explicit missing category |
| Sex | Administrative sex | Female, male, or missing category |
| Length of stay | Discharge date minus admission date in source extract | Capped at the 2017 99th percentile (54 days), log1p transformed, median-imputed |
| Principal diagnosis group | Source diagnosis-group label | Harmonized to circulatory, digestive, endocrine, genitourinary, infection, musculoskeletal/injury, neoplasm, neurologic, other, planned, respiratory, or skin |
| Discharge destination | Destination indicators plus disposition code | Home, facility, acute transfer, community service, other, or missing |
| Prior utilization field | Putative recent same-condition readmission field | Not modeled: blank in 40,931/40,971 episodes (99.9%) |

# Table S3. Model Development and Reproducibility Details

| **Item** | **Specification** |
| --- | --- |
| Unit of analysis | Discharge episode; repeated episodes treated independently because patient identifiers were unavailable |
| Development-validation split | Calendar-year split: 2017 development (n=19,890); 2018 temporal validation (n=21,081) |
| Categorical preprocessing | Explicit missing categories, most-frequent imputation if required by pipeline, one-hot encoding, unknown categories ignored, encoder min_frequency=5 |
| Continuous preprocessing | Length of stay capped at 54 days, log1p transformed, median-imputed, scaled without centering |
| Logistic regression | C=0.1; liblinear solver; max_iter=3000 |
| Random forest | 400 trees; unrestricted depth; min_samples_leaf=20; max_features=sqrt |
| Extreme gradient boosting | 350 estimators; max_depth=3; learning_rate=0.03; subsample=0.8; colsample_bytree=0.8; min_child_weight=5; L2 lambda=2 |
| Internal resampling | Five-fold stratified cross-validation in 2017; seed 20260801 |
| Probability calibration | Platt scaling fitted to 2017 out-of-fold predictions and applied to the model refitted on all 2017 episodes |
| Model selection | Highest 2017 out-of-fold AUPRC; Brier score and AUROC secondary |
| Confidence intervals | 1000 stratified nonparametric percentile bootstrap replicates in 2018 |
| Software | Python 3.13.5; NumPy 2.3.5; scikit-learn 1.8.0; XGBoost 3.1.3; statsmodels 0.14.6; SciPy 1.17.0 |

# Table S4. Temporal-Validation Performance and Calibration

| **Model** | **AUROC (95% CI)** | **AUPRC (95% CI)** | **Brier (95% CI)** | **Intercept** | **Slope** | **O:E** |
| --- | --- | --- | --- | --- | --- | --- |
| Random forest | 0.816 (0.809-0.823) | 0.593 (0.579-0.607) | 0.119 (0.116-0.121) | -0.117 | 0.974 | 0.951 |
| XGBoost | 0.817 (0.810-0.824) | 0.589 (0.575-0.604) | 0.119 (0.116-0.121) | -0.119 | 0.976 | 0.948 |
| Logistic regression | 0.806 (0.798-0.813) | 0.541 (0.528-0.557) | 0.121 (0.119-0.124) | -0.107 | 0.974 | 0.956 |

**Note:** Calibration intercept 0, slope 1, and O:E ratio 1 indicate ideal calibration. Mean predicted risk was 21.8% for random forest versus an observed 20.7%.

# Figure S1. Calibration Plot

Figure S1. Decile-based calibration in the 2018 temporal-validation cohort after Platt scaling based on 2017 out-of-fold predictions.

# Figure S2. Decision-Curve Analysis

Figure S2. Random-forest decision-curve net benefit compared with treat-all and treat-none strategies across thresholds from 0.05 to 0.50. This retrospective analysis does not demonstrate benefit from actual clinical implementation.

# Table S5. Development-Derived Risk Tiers Applied to 2018

| **Tier** | **Score interval** | **N** | **Share** | **Events** | **Observed rate** |
| --- | --- | --- | --- | --- | --- |
| Low | <0.1256 | 10,120 | 48.0% | 661 | 6.5% |
| Moderate | 0.1256 to <0.3014 | 6,271 | 29.7% | 1,160 | 18.5% |
| High | 0.3014 to <0.7746 | 3,739 | 17.7% | 1,769 | 47.3% |
| Very high | >=0.7746 | 951 | 4.5% | 771 | 81.1% |

# Table S6. Random-Forest Feature Importance

| **Feature** | **Aggregated impurity importance** |
| --- | --- |
| Principal diagnosis group | 84.6% |
| Length of stay | 8.6% |
| Age band | 4.5% |
| Sex | 1.2% |
| Discharge destination group | 1.2% |

**Note:** Importance is descriptive and not causal. Within diagnosis group, the planned admission/discharge category alone accounted for 62.5% of total model importance.

# Table S7. Selected Subgroup Performance

| **Domain** | **Group** | **Events/N (%)** | **AUROC** | **AUPRC** | **Brier** | **O:E** | **Slope** |
| --- | --- | --- | --- | --- | --- | --- | --- |
| Age | <65 | 2290/10670 (21.5%) | 0.861 | 0.662 | 0.107 | 0.966 | 0.980 |
| Age | >=65 | 2071/10410 (19.9%) | 0.751 | 0.497 | 0.131 | 0.935 | 0.960 |
| Sex | female | 1846/8999 (20.5%) | 0.827 | 0.611 | 0.113 | 0.932 | 1.000 |
| Sex | male | 2515/12082 (20.8%) | 0.807 | 0.583 | 0.123 | 0.965 | 0.953 |
| Discharge destination | facility | 271/1190 (22.8%) | 0.539 | 0.262 | 0.178 | 0.974 | 0.411 |
| Discharge destination | home | 3835/18537 (20.7%) | 0.825 | 0.606 | 0.116 | 0.954 | 0.975 |
| Discharge destination | missing | 252/1305 (19.3%) | 0.821 | 0.627 | 0.106 | 0.899 | 1.083 |

**Note:** Subgroup analyses are descriptive and were not adjusted for multiple comparisons. Facility and small destination categories showed unstable discrimination; categories with insufficient events were omitted.

# Table S8. Diagnosis-Group Performance

| **Diagnosis group** | **N** | **Events (%)** | **AUROC** | **AUPRC** | **O:E** |
| --- | --- | --- | --- | --- | --- |
| circulatory | 2,364 | 189 (8.0%) | 0.671 | 0.142 | 0.902 |
| digestive | 2,590 | 282 (10.9%) | 0.679 | 0.178 | 1.131 |
| endocrine | 287 | 40 (13.9%) | 0.537 | 0.213 | 1.039 |
| genitourinary | 2,087 | 242 (11.6%) | 0.679 | 0.203 | 0.853 |
| infection | 584 | 87 (14.9%) | 0.690 | 0.249 | 0.910 |
| musculoskeletal_injury | 3,195 | 217 (6.8%) | 0.696 | 0.138 | 0.961 |
| neoplasm | 3,050 | 944 (31.0%) | 0.578 | 0.365 | 0.918 |
| neurologic | 234 | 20 (8.5%) | 0.637 | 0.193 | 0.727 |
| other | 1,610 | 113 (7.0%) | 0.645 | 0.137 | 0.783 |
| planned | 2,411 | 1,783 (74.0%) | 0.608 | 0.794 | 0.969 |
| respiratory | 2,034 | 366 (18.0%) | 0.594 | 0.218 | 0.979 |
| skin | 635 | 78 (12.3%) | 0.675 | 0.220 | 1.109 |

# Table S9. Sensitivity Analyses for the Selected Random Forest

| **Analysis** | **N** | **Events (%)** | **AUROC** | **AUPRC** | **Brier** |
| --- | --- | --- | --- | --- | --- |
| Exclude post-discharge death within 30 days | 20,966 | 4,310 (20.6%) | 0.817 | 0.594 | 0.118 |
| Composite readmission or death within 30 days | 21,081 | 4,425 (21.0%) | 0.816 | 0.594 | 0.120 |
| Age >=18 years | 20,811 | 4,352 (20.9%) | 0.814 | 0.593 | 0.120 |
| Exclude planned index diagnosis (fixed full-cohort model) | 18,670 | 2,578 (13.8%) | 0.726 | 0.283 | 0.110 |
| Exclude planned index diagnosis (refitted model) | 18,670 | 2,578 (13.8%) | 0.726 | 0.284 | 0.110 |

Note: Both analyses exclude 2018 index episodes in the planned admission/discharge diagnosis category. The fixed full-cohort model retains the model and calibration fitted using all eligible 2017 episodes; the refitted model re-estimates both using the corresponding restricted 2017 subset. The two approaches are evaluated in the same 18,670 validation episodes but need not produce identical predictions or AUPRCs, even when AUROC is identical after rounding to three decimals. This index-diagnosis exclusion is an imperfect proxy for potentially planned readmissions and does not directly identify or remove all planned subsequent admissions.

# Table S10. Historical Nursing Cohort Definitions and Provenance

| **Dataset** | **N** | **Purpose** | **Provenance interpretation** |
| --- | --- | --- | --- |
| Shared decision-making | 72 cases | Structured discharge-direction discussion | Matches the previously published SDM arm in sample size and key outcomes [13]; treated as overlapping, not independent |
| Long-term care linkage | 364 cases | Service uptake and time to service initiation | Matches the previously published comparison arm [13]; treated as overlapping, not independent |
| Remote monitoring | 43 patients | Paired utilization before and after enrollment at 3, 6, and 12 months | Enrollment dates November 12, 2020-November 10, 2021; appears updated/overlapping with [11], but crosswalk unavailable |

# Table S11. Remote-Monitoring Paired Outcomes

| **Window** | **Outcome** | **Before mean** | **After mean** | **Mean change** | **Wilcoxon P** |
| --- | --- | --- | --- | --- | --- |
| 3 months | Outpatient visits | 0.88 | 1.60 | +0.72 | P=0.015 |
| 3 months | Emergency visits | 0.65 | 0.19 | -0.47 | P=0.001 |
| 3 months | Readmissions | 0.58 | 0.28 | -0.30 | P=0.016 |
| 3 months | Hospital days | 9.49 | 3.51 | -5.98 | P=0.019 |
| 6 months | Outpatient visits | 1.40 | 2.60 | +1.21 | P=0.015 |
| 6 months | Emergency visits | 0.77 | 0.40 | -0.37 | P=0.030 |
| 6 months | Readmissions | 0.70 | 0.42 | -0.28 | P=0.021 |
| 6 months | Hospital days | 10.58 | 8.70 | -1.88 | P=0.284 |
| 12 months | Outpatient visits | 2.23 | 4.02 | +1.79 | P=0.010 |
| 12 months | Emergency visits | 1.02 | 0.67 | -0.35 | P=0.063 |
| 12 months | Readmissions | 0.95 | 0.60 | -0.35 | P=0.024 |
| 12 months | Hospital days | 12.67 | 9.09 | -3.58 | P=0.147 |

**Note:** Two-sided Wilcoxon signed-rank tests used the wilcox zero-difference method. No multiplicity adjustment was applied; all P values are exploratory. The cohort had no concurrent control group.

# Table S12. Proposed Silent-Run Monitoring and Recalibration Dashboard

| **Domain** | **Metric** | **Frequency** | **Potential action** |
| --- | --- | --- | --- |
| Data readiness | Valid score generation; data latency; predictor missingness | Daily/monthly | Repair interfaces or revise missingness handling |
| Discrimination | AUROC and AUPRC | Quarterly | Investigate drift; consider model revision |
| Calibration | Brier score, intercept, slope, O:E, calibration plot | Quarterly | Intercept update or probability recalibration |
| Risk tiers | Observed event rate and volume by tier | Quarterly | Revise thresholds against capacity and event rates |
| Equity | Calibration and action rates by age, sex, diagnosis, destination | Quarterly | Investigate disparities; modify workflow or model |
| Nursing workload | Review time, alerts per nurse, override rate | Monthly | Adjust presentation or thresholds |
| Care process | Case-management review, SDM, referral, follow-up completion | Monthly | Identify workflow bottlenecks |
| Patient outcomes | 30-day readmission and ED visits; 90-day hospital days | Quarterly | Proceed, recalibrate, revise, or stop |

# Table S13. Historical Cohort Descriptive Outcomes

| **Dataset** | **Outcome** | **Result** |
| --- | --- | --- |
| Shared decision-making | Discharge direction changed | 30/72 (42%) |
| Shared decision-making | Recommended and actual direction concordant | 43/72 (60%) |
| Shared decision-making | Any 30-day readmission | 11/72 (15%) |
| Shared decision-making | 3-month survival | 64/72 (89%) |
| Long-term care | Any 30-day readmission | 65/364 (17.9%) |
| Long-term care | 3-month survival | 330/364 (90.7%) |
| Long-term care linkage | Used service | 301/364 (82.7%) |
| Long-term care linkage | Valid nonnegative service dates among users | 297/301 (98.7%) |
| Long-term care linkage | Started within 3 days | 198/297 (66.7%) |
| Long-term care linkage | Started within 7 days | 268/297 (90.2%) |
| Long-term care linkage | Time to service start | Median 1 day (IQR 0-5) |
